# Gen Z, Gender, and COVID-19

**DOI:** 10.1017/S1743923X20000434

**Published:** 2020-07-09

**Authors:** Melissa Deckman, Jared McDonald, Stella Rouse, Mileah Kromer

**Affiliations:** 1 Washington College; 2 Stanford University; 3 University of Maryland; 4 Goucher College

**Keywords:** gender gap, Generation Z, coronavirus, party polarization

## Abstract

Using a national survey of Generation Z conducted in late May 2020, we measure attitudes about the impact of the coronavirus on personal health, financial and job concerns, views about shelter-in-place laws, and 2020 voting intentions. Gen Z women express greater health and economic concerns and support for shelter-in-place measures than their male counterparts, but this gender gap is largely mitigated by party and other covariates. Party also mediates the differences between young male and female voters concerning the influence of the coronavirus on their vote choice in 2020. Notably, women have significantly greater concern about the impact of COVID-19 on their personal financial situation, while Gen Z men express more concern about their personal health amid COVID-19 in more fully specified statistical models. This research contributes to the growing literature that examines not only the sorting effect of party on the gender gap but also how different identities—in this case, generation—can help explain the persistent political divides between men and women.

Political scientists have long studied the American gender gap in public opinion, finding that women tend to be more liberal than men (Lizotte 2020). Yet women are far from monolithic in their political views, and thus the interplay of ideology and gender in the current era has largely been mediated by elite-level party sorting (Gillion, Ladd, and Meredith 2018). Women and men have largely “sorted” themselves into the parties that best match their policy preferences (men with the Republican Party and women with the Democratic Party). Most research on the partisan gender gap examines men and women solely through a gender lens, paying less attention to other identities that may help explain the divide, with some notable exceptions (cf. Junn and Masuoka 2019).

For example, we know little about the strength of the gender gap in public opinion among the youngest generation of voters: Generation Z (defined as those born after 1996). The lack of research on this topic is problematic when we consider recent developments in American politics. Following the 2016 election of Donald Trump, women mounted a large-scale political resistance to his presidency, highlighted by the 2017 Women's March. In 2018, a record number of Democratic women sought elected office, suggesting a political awakening for young women. Recent scholarship affirms what these events show: unlike prior generations, Gen Z women are more engaged and enthusiastic about politics than Gen Z men (Campbell and Wolbrecht 2019; Vandermaas et al. 2018). Given that Gen Z women already exhibit some different political behaviors than Gen Z men, an analysis that considers gender differences in public opinion among this nascent generation is warranted.

In this article, we examine whether and to what extent gender gaps emerge among Gen Z with respect to concerns about the novel coronavirus and the government's response to it, and whether partisanship mitigates those attitudes. We examine the gender gap among Gen Z Americans on the topic of COVID-19 because of the unique challenges that the pandemic poses for the youngest Americans. Just as the economic recession of 2008 damaged the long-term career expectations of millennials (de Hauw and de Vos 2010), members of Gen Z may feel the effects of the COVID-19 economic downturn for decades to come. At best, this generation faces an uncertain future, and their attitudes reflect that they have already been hit particularly hard by the pandemic (Parker and Igielnik 2020). A recent national survey conducted by the Pew Research Center (Schaeffer and Rainie 2020) suggests that younger Americans, more so than their older counterparts, view the coronavirus as stress inducing and a greater threat to their personal finances. Moreover, Pickup, Stecula, and van der Linden (2020) find that partisanship shapes attitudes about the coronavirus, with Democrats expressing more concern than Republicans about the pandemic, while also being less confident in the federal government's handling of it.

Given that members of Gen Z are less likely to identify with a political party than older Americans (CIRCLE 2018; Young 2019), considering how partisanship shapes attitudes about COVID-19 is particularly imperative. In light of this, we examine the extent to which gender and party shape Gen Z concerns about the impact of the coronavirus on their own health, financial and job concerns, and their views about shelter-in-place laws, as well as the extent to which the coronavirus will shape their voting intentions in the 2020 presidential election.

## GENERATION Z AND GENDER GAPS IN POLITICAL OPINION

On a wide range of public policy issues, the preferences of women and men differ. Women are less likely than men to support the use of force, while being more likely to oppose capital punishment and support gun control (Caughell 2016; Celinska 2007; Haider-Marker and Joslyn 2001; Norrander 2008). Women are also more likely to support greater social welfare spending and a more activist government role in assisting the poor (Caughell 2016; Fox and Oxley 2015; Lizotte 2020; Norrander 2008). There is also evidence of a gender gap in attitudes toward the coronavirus specifically. During the first months of the outbreak, women were more likely to support government steps to combat the virus and to take personal measures such as washing their hands more often or avoiding physical contact (Kahn 2020).

Explanations for the gender gap in public opinion vary, but some scholars argue that the divergence may be linked to gender role socialization (Diekman and Schneider 2010). Given that women are often socialized to be more compassionate and nurturing, they are primed to hold more liberal positions on social compassion issues and to support a larger government role on issues such as health care and school spending (Eagly and Diekman 2006; Greenlee 2014). Women's greater empathy for the poor may also lead them to support more social welfare spending (Huddy, Cassese, and Lizotte 2008). Economic theories of the gender gap posit that because women are more economically vulnerable than men, and more likely to receive economic benefits from the government, they are more supportive of government social spending (Norrander 2008). Mary-Kate Lizotte (2020, 31) argues that such gender differences can be best understood through a values perspective, specifically the idea that “women appear to have a greater concern for the well-being of others.” In turn, these “pro-social values” lead women to hold more liberal positions on a range of issues (34)

We hypothesize several scenarios in terms of how gender may influence Gen Z's attitudes about the coronavirus in our present study. First, because of gender socialization, women's economic vulnerability, and Lizotte's (2020) pro-social values perspective, Gen Z women will be more likely to support government intervention in handling COVID-19, to express more concern about the financial impact of the virus, and to express more concern about the virus's impact on their families. They may also be more likely to consider responses to COVID-19 in their vote choice. Second, concerns for one's own health related to the pandemic might be more prevalent among Gen Z men because men are less empathetic to the needs of others; similarly, that ethos may also result in Gen Z men being less likely to support shelter-in-place policies than Gen Z women. Lastly, it may be more likely instead that on most measures, partisanship exerts a stronger influence on attitudes about the coronavirus, showing that Gen Z is not immune to partisan pressures.

## DATA AND MEASUREMENT

We conducted a nationally representative survey of 1,049 American adults aged 18–24 (members of Generation Z) using a Qualtrics online panel from May 19 to May 28, 2020. Our analysis includes 1,008 responses from Gen Zers who identify as cis female (*n* = 506) or cis male (*n* = 502).^1^ The survey was fielded after “shelter-in-place” measures had been imposed by most U.S. states, after Congress had passed a large stimulus package to address the economic impact of COVID-19, and as deaths due to COVID-19 neared 100,000. Thus, respondents were answering our survey when the coronavirus was dominating public discourse.

Gen Zers were asked how concerned they were about the impact of COVID-19 on a variety of issues, their attitudes toward the shelter-in-place measures, and whether the response to COVID-19 was important to their vote choice. Tables A1–A3 in the online appendix contain the question wording of the items used in our analyses and the response distributions. For ease of interpretation, the results presented here collapse all 3-point scales to 2, though logit models using all response options (found in Appendix B) do not change the conclusions.

## FINDINGS

**Figure 1.**
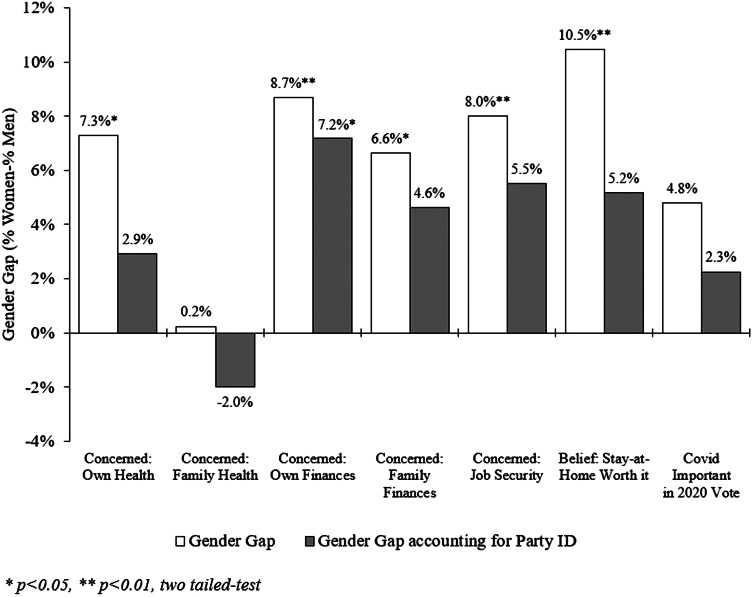
Gender Gap on COVID-19 Opinions, with and without Accounting for Partisanship

Figure 1 displays the results for Gen Z women and men who stated that they were somewhat or very concerned across five health and economic COVID-19 related items, as well as the percentage of respondents who said that strict shelter-in-place measures had been worth it to minimize the spread of the coronavirus. It also displays the results for the importance of COVID-19 response by government as a factor in respondents’ vote choice in 2020. The lighter shaded bars show the percent response difference between women and men (i.e., the results of a bivariate regression). These results reveal that Gen Z women express greater health and economic concerns about COVID-19 than Gen Z men. The gender gap ranges from 6.6 to 8.7 percentage points, and the differences are statistically significant for all but two items (concern for family's health and whether COVID-19 factors into 2020 vote choice). Similarly, Gen Z women are more likely than Gen Z men to believe that the stay-at-home orders were worth it; the 10.5 percentage point difference is also statistically significant.

The story changes quite a bit when we account for partisanship. The darker bars in Figure 1 present the same results as the lighter bars, but with a single control variable for partisanship (on a 7-point scale). Partisanship reduces the gender gap across all COVID-19 response items, and the gender differences are now statistically significant for only one item—concern for personal financial situation. The ordered logit and logit results (presented in Tables B1, B2, and B3 in Appendix B) largely confirm these findings.^2^ Accounting for other covariates that may affect COVID-19 concerns (e.g., race, education, and income), we find that partisanship absorbs a large amount of explanatory value^3^; as Gen Z respondents become more Republican, they become less concerned about the impacts of the coronavirus. Republicans are also less likely to say that shelter-in-place laws are worth it. However, partisanship does not completely erase the gender gap in two cases: Gen Z women are more likely to be concerned that COVID-19 will harm their ability to keep their jobs. We also find that Gen Z men are significantly more likely to express concerns about their own personal health with respect to COVID-19, potentially because concern for one's own health does not require the empathic response described by Lizotte (2020).

Our final analysis examines how the government's response to the COVID-19 pandemic may affect Gen Zers’ vote in the 2020 election. Once again, we find that while Gen Z women are more likely than Gen Z men to say that the response to the pandemic is a very or somewhat important factor in their vote, this gap is strongly mediated by partisanship. The ordered logit results (Table B3 in Appendix B) indicate a statistically significant effect for partisanship in the presence of other potential confounding factors, with Republicans expressing far less concern about COVID as a factor in their vote this fall. It is likely that gender will play a role in the 2020 election—even among the youngest Americans—but Gen Z women and men have largely sorted themselves into the parties that best represent their concerns and priorities on a wide range of issues, including those related to COVID-19.

## DISCUSSION

Our study, which considers how gender influences attitudes about the coronavirus, is among the first to examine gender gaps in public opinion among Generation Z. At first glance, Gen Z women express more concern about many of the virus's implications and more support for shelter-in-place laws, which is consistent with much of the scholarly literature on the gender gap that links concerns about care issues with women's social values, economic vulnerability, and gender socialization. However, most gender differences with regard to the coronavirus fail to emerge as significant after controlling for partisanship and other covariates. Notably, though, the two significant gender differences that emerge in our multivariate analysis do speak to the gender gap literature: women's greater concern about how COVID-19 may jeopardize their employment echoes earlier studies that argue that the gender gap is rooted in women's greater economic vulnerability. Men's greater prioritization of their own personal health in wake of COVID-19 may also speak to their lack of empathy or “pro-social values” (Lizotte 2020).

No large gender gaps emerge with respect to how Gen Z will factor responses to COVID into their votes in the 2020 election, though it should be noted that 58% of all Gen Z Americans say this issue is very important to their vote choice; an additional 30% say it is somewhat important. Overall, however, while gender may matter to these opinions even among the youngest cohort of Americans, its influence is largely mediated by parties, which play a crucial role in sorting the attitudes and policy preferences of men and women of older generations.

## Footnotes

1. Because of the small number of respondents who identified outside of the gender binary, we omitted those responses.

2. Table B1 in the online appendix provides the ordered logit results for the first six items described in Figure 1. Table B2 provides a logit model predicting the belief that the stay-at-home orders were worth it. Finally, Table B3 provides an ordered logit model for the importance that respondents put on COVID-19 response for their 2020 vote choice.

3. Because our sample has a slight overrepresentation of 18-year-olds (see Table A4 in the appendix), we included a control for age of respondent out of an abundance of caution. Income refers to self-reported family income; education is an ordinal measure ranging from 1 (less than high school) to 8 (professional or doctoral degree).

## Supplementary material

For supplementary material accompanying this paper visit http://dx.doi.org/10.1017/S1743923X20000434. click here to view supplementary material
