# Supplementary material for: Gen Z, Gender, and COVID-19
Source: Politics & Gender. 2020 Jul 9:1–9. doi: 10.1017/S1743923X20000434 (PMC7443560; doi:10.1017/S1743923X20000434)
Supplement: Supplementary file 1 [file S1743923X20000434sup001.docx]

**Appendix A**

**Table A1: Dependent Variables; Concern over Impact of COVID-19 (in percent)**

*Question: How concerned are you about the impact of COVID-19 (Coronavirus) on the following:*

|  | Very | Somewhat | Not at all | Unsure |
| --- | --- | --- | --- | --- |
| Your own health | 31 | 39 | 28 | 2 |
| The health of your family | 52 | 34 | 12 | 2 |
| Your personal financial situation | 40 | 33 | 24 | 3 |
| The financial situation of your family | 44 | 31 | 21 | 4 |
| The ability to get or keep a job | 44 | 29 | 24 | 4 |
|  |  |  |  |  |

*n* = 1,008

**Table A2: Dependent Variable; Attitudes toward Shelter-in-Place Measures (in percent)**

*Question: Which of the following statements comes closest to your view, even if neither is perfect with respect to shelter-in-place measures and the Coronavirus:*

|  | Percent |
| --- | --- |
| Strict shelter-in-place measures are worth it in order to protect people and limit the spread of coronavirus. | 61 |
| Strict shelter-in-place measures are placing unnecessary burdens on people and the economy and are causing more harm than good. | 24 |
| Unsure | 15 |

*n* = 1,008

**Table A3: Dependent Variable; Impact of COVID-19 Response on Vote Choice (in percent)**

*Question: The following is a list of issues and concerns that might influence people's vote choices in November. For each one, please tell me whether it will be very important, somewhat important or not important at all in determining your vote: “The response to the COVID-19 pandemic.”*

|  | Percent |
| --- | --- |
| Very Important | 58 |
| Somewhat Important | 30 |
| Not at all Important | 7 |
| Unsure | 5 |

*n* = 1,008

**Table A4: Sample Demographics for Full Survey, Unweighted and Weighted**

| **SAMPLE DEMOGRAPHICS** | | **Unweighted Frequency** | **Weighted Frequency** |
| --- | --- | --- | --- |
| ***Age*** | 18 | 19.6% | 19.9% |
|  | 19 | 16.4% | 17.2% |
|  | 20 | 16.4% | 16.4% |
|  | 21 | 15.0% | 14.7% |
|  | 22 | 11.4% | 11.2% |
|  | 23 | 9.3% | 9.1% |
|  | 24 | 11.9% | 11.5% |
|  | White | 61.1% | 52.0% |
| ***Race*** | Black | 17.7% | 14.0% |
|  | Other | 22.2% | 34.0% |
|  | Male | 51.3% | 47.9% |
| ***Gender*** | Female | 44.7% | 48.3% |
|  | Trans/Non-Conforming | 4.0% | 3.9% |
| ***Household Income*** | <$50k | 55.3% | 50.0% |
|  | $50k-$100k | 25.5% | 28.8% |
|  | >$100k | 19.2% | 21.2% |
| ***Education*** | High School or Below | 38.17% | 36.64% |
|  | Some College | 34.29% | 35.23% |
|  | College or Advanced Degree | 26.84% | 28.13% |
| ***Partisanship*** | Democrat/Lean Democrat | 49.8% | 52.0% |
|  | Republican/Lean Republican | 28.5% | 27.0% |
|  | Independent/Do Not Lean | 21.7% | 21.0% |

**Appendix B**

| **Table B1: Ordered Logit – Predicting COVID-19 Concern for…** | | | | | | | |
| --- | --- | --- | --- | --- | --- | --- | --- |
|  | | | | |  | |  |
|  | Own Health | Family Health | Own Finances | Family Finances | | Ability to Find/Keep Job | |
|  | (1) | (2) | (3) | (4) | | (5) | |
|  | |  | |  | | | |
| Gender (Male) | 0.273* | -0.054 | -0.136 | -0.025 | | -0.282* | |
|  | (0.137) | (0.143) | (0.138) | (0.138) | | (0.140) | |
|  |  |  |  |  | |  | |
| Partisanship | -0.143** | -0.174** | -0.066* | -0.070* | | -0.121** | |
|  | (0.034) | (0.033) | (0.034) | (0.034) | | (0.032) | |
|  |  |  |  |  | |  | |
| Race (White) | -0.451** | -0.156 | -0.009 | -0.245 | | 0.144 | |
|  | (0.171) | (0.178) | (0.163) | (0.171) | | (0.170) | |
|  |  |  |  |  | |  | |
| Race (Black) | 0.645** | -0.260 | 0.346 | 0.023 | | -0.179 | |
|  | (0.200) | (0.023) | (0.209) | (0.202) | | (0.204) | |
|  |  |  |  |  | |  | |
| Family Income | -0.075** | -0.028 | -0.098** | -0.105** | | -0.046 | |
|  | (0.025) | (0.026) | (0.024) | (0.025) | | (0.024) | |
|  |  |  |  |  | |  | |
| Education | 0.094** | 0.057 | 0.069 | 0.045 | | 0.048 | |
|  | (0.056) | (0.057) | (0.053) | (0.052) | | (0.056) | |
|  |  |  |  |  | |  | |
| Age | 0.047 | 0.019 | 0.058 | 0.008 | | 0.007 | |
|  | (0.039) | (0.039) | (0.036) | (0.036) | | (0.037) | |
|  |  |  |  |  | |  | |
| Cut 1 | -0.587 | -2.340 | -0.499 | -1.940 | | -1.575 | |
|  | (0.765) | (0.770) | (0.746) | (0.747) | | (0.755) | |
|  |  |  |  |  | |  | |
| Cut 2 | 1.226 | -0.436 | 1.010 | -0.461 | | -0.253 | |
|  | (0.764) | (0.773) | (0.745) | (0.746) | | (0.754) | |
|  | |  | |  | | | |
| Observations | 982 | 986 | 972 | 958 | | 970 | |
| R^2^ | 0.045 | 0.023 | 0.023 | 0.020 | | 0.014 | |
|  | |  | |  | | | |
| *Notes: Robust standard errors in parentheses. * denotes p<0.05, two-tailed, ** denotes p<0.01, two tailed.* | | | | | | | |

| \| **Table B2: Logit – Predicting Belief that Shelter-in-Place Orders are Worth it** \| \| \| --- \| --- \| \| Gender (Male) \| 0.250 \| \| \|  \| (0.186) \| \| \|  \|  \| \| \| Partisanship \| -0.272** \| \| \|  \| (0.041) \| \| \|  \|  \| \| \| Race (White) \| -0.214 \| \| \|  \| (0.225) \| \| \|  \|  \| \| \| Race (Black) \| 0.387 \| \| \|  \| (0.271) \| \| \|  \|  \| \| \| Family Income \| 0.025 \| \| \|  \| (0.032) \| \| \|  \|  \| \| \| Education \| 0.033 \| \| \|  \| (0.068) \| \| \|  \|  \| \| \| Age \| -0.015 \| \| \|  \| (0.044) \| \| \|  \|  \| \| \| Constant \| 2.266 \| \| \|  \| (0.859) \| \| \|  \| \| \| \| Observations \| 848 \| \| \| R^2^ \| 0.066 \| \| \| *Notes: Robust standard errors in parentheses. * denotes p<0.05, two-tailed, ** denotes p<0.01, two tailed.* \| \|   **Table B3: Ordered Logit – Predicting Importance of COVID-19 on 2020 Vote Choice** | |  |
| --- | --- | --- | --- | --- | --- | --- | --- | --- | --- | --- | --- | --- | --- | --- | --- | --- | --- | --- | --- | --- | --- | --- | --- | --- | --- | --- | --- | --- | --- | --- | --- | --- | --- | --- | --- | --- | --- | --- | --- | --- | --- | --- | --- | --- | --- | --- | --- | --- | --- | --- | --- | --- | --- | --- | --- | --- | --- | --- | --- | --- | --- | --- | --- | --- | --- | --- | --- | --- | --- | --- | --- | --- | --- | --- | --- | --- | --- | --- | --- | --- | --- | --- | --- | --- |
| Gender (Male) | -0.076 | |
|  | (0.151) | |
|  |  | |
| Partisanship | -0.121** | |
|  | (0.033) | |
|  |  | |
| Race (White) | 0.032 | |
|  | (0.173) | |
|  |  | |
| Race (Black) | 0.087 | |
|  | (0.221) | |
|  |  | |
| Family Income | -0.049 | |
|  | (0.026) | |
|  |  | |
| Education | 0.047 | |
|  | (0.063) | |
|  |  | |
| Age | 0.050 | |
|  | (0.043) | |
|  |  | |
| Cut 1 | -2.033 | |
|  | (0.840) | |
|  |  | |
| Cut 2 | 0.037 | |
|  | (0.841) | |
|  | | |
| Observations | 960 | |
| R^2^ | 0.016 | |
| *Notes: Robust standard errors in parentheses. * denotes p<0.05, two-tailed, ** denotes p<0.01, two tailed.* | |  |
